# Forensic analysis and sequence variation of 133 STRs in the Hakka population

**DOI:** 10.3389/fgene.2024.1347868

**Published:** 2024-01-22

**Authors:** Yuhang Feng, Yutao Zhao, Xiaoyu Lu, Haiyan Li, Kai Zhao, Meisen Shi, Shaoqing Wen

**Affiliations:** ^1^ MOE Key Laboratory of Contemporary Anthropology, School of Life Sciences, Fudan University, Shanghai, China; ^2^ Public Security Bureau of Zhaoqing Municipality, Zhaoqing, China; ^3^ Deepreads Biotech Company Limited, Guangzhou, China; ^4^ Criminal Technology Center of Guangdong Provincial Public Security Department, Guangzhou, China; ^5^ Criminal Justice College of China University of Political Science and Law, Beijing, China; ^6^ Institute of Archaeological Science, Fudan University, Shanghai, China; ^7^ MOE Laboratory for National Development and Intelligent Governance, Fudan University, Shanghai, China

**Keywords:** STR, Y-STR, A-STR, massively parallel sequencing, forensic genetics, Meizhou Hakka population, sequence variation

## Abstract

**Introduction:** Short Tandem Repeats (STRs) are highly valuable genetic markers in forensic science. However, the conventional PCR-CE technique has limitations, and the emergence of massively parallel sequencing (MPS) technology presents new opportunities for STR analysis. Yet, there is limited research on Chinese population diversity using MPS.

**Methods:** In this study, we obtained genotype data for 52 A-STRs and 81 Y-STRs from the Hakka population in Meizhou, Guangdong, China, using the Forensic Analysis System Multiplecues SetB Kit on the MGISEQ-2000 platform.

**Results:** Our findings demonstrate that these 133 STRs are highly efficient for forensic applications within the Meizhou Hakka population. Statistical analysis revealed H_obs_ values ranging from 0.61306 to 0.91083 and H_exp_ values ranging from 0.59156 to 0.91497 for A-STRs based on length polymorphism. For sequence polymorphism, H_obs_ values ranged from 0.61306 to 0.94586, and H_exp_ values fluctuated between 0.59156 and 0.94487. The CPE values were 1-5.0779620E-21 and 1-3.257436E-24 for length and sequence polymorphism, respectively, while the CPD values were 1-1.727007E-59 and 1-5.517015E-66, respectively. Among the 80 Y-STR loci, the HD values for length and sequence polymorphism were 0.99764282 and 0.99894195, respectively. The HMP values stood at 0.00418102 and 0.00288427, respectively, and the DC values were 0.75502742 and 0.83363803, respectively. For the 52 A-STR loci, we identified 554 and 989 distinct alleles based on length and sequence polymorphisms, respectively. For the 81 Y-STR loci, 464 and 652 unique alleles were detected at the length and sequence level, respectively. Population genetic analysis revealed that the Meizhou Hakka population has a close kinship relationship with the Asian populations THI and KOR based on length polymorphism data of A-STRs. Conversely, based on length polymorphism data of Y-STRs, the Meizhou Hakka population has the closest kinship relationship with the Henan Han population.

**Discussion:** Overall, the variation information of repeat region sequences significantly enhances the forensic identification efficacy of STR genetic markers, providing an essential database for forensic individual and paternity testing in this region. Additionally, the data generated by our study will serve as a vital resource for research into the genetic structure and historical origins of the Meizhou Hakka population.

## 1 Introduction

Short tandem repeats (STRs) are DNA sequences made up of repeating units of two to six base pairs. These STRs are highly polymorphic and play a critical role in individual and kinship identification in forensic science (Fan and Chu, 2007). The most common method for genotyping STRs has been through polymerase chain reaction (PCR) and capillary electrophoresis (CE) (Butler et al., 2004; Shewale et al., 2012). However, CE has limitations in terms of the number of STRs it can detect simultaneously and its inability to account for sequence alterations within the repeated region (Gill et al., 1997). These limitations can hinder the efficiency of identifying specific kinship relationships and may lead to errors in lineage searches. With advancements in technology, massively parallel sequencing (MPS) has become widely used in forensic science (Guo et al., 2016; Zhou et al., 2016; Guo et al., 2017; Pereira et al., 2017; Feng et al., 2022). MPS offers several advantages over traditional PCR-CE, including the ability to analyze a larger number of samples and incorporate multiple genetic markers into a complex panel. It also provides more detailed information about nucleotide sequences, allowing for better distinction and accuracy. However, the length features of STR genetic markers can pose challenges for conventional MPS sequencing strategies (Phillips, 2017). To overcome this limitation, long-read sequencing platforms are needed to ensure comprehensive and accurate capture of STR genotype data, particularly in forensic genetics. Accurate calculation of allele frequencies in the relevant population is crucial for determining the random match probability between DNA found at a crime scene and that of a suspect (Butler, 2015). While there is a wealth of high-quality genetic data for STRs based on length polymorphism, our understanding of STRs based on sequence polymorphism is still incomplete. Therefore, the establishment of a sequence-based STR database is essential to assist forensic professionals in their application to cases.

Meizhou, located in the northeastern region of Guangdong Province in China, is known as the “World Hakka Capital”. The Hakka people, a subpopulation of the Han Chinese, speak the Hakka dialect. They are believed to have originated in northern China and migrated southward over various historical periods (Wu, 1995; Zhang, 1997). Today, approximately 60% of the Hakka population is concentrated in the Meizhou region, where they have preserved the ancient Han culture and incorporated the indigenous culture of southern China (Tan, 2008). The Hakka people have developed a unique culture due to their prolonged habitation in mountainous regions, earning them the nickname “mountain-dwelling ethnic group”. The Meizhou Hakka possess a wealth of genetic information resources, making them an ideal population for genetic analysis in forensic genetics.

In this study, we utilized the MPS platform and the Forensic Analysis System Multiplecues SetB Kit to evaluate the allele frequencies and forensic-related parameters of 52 autosomal STRs (A-STRs) and 81 Y-chromosome STRs (Y-STRs) based on length and sequence polymorphism in 628 Hakka individuals from Meizhou, Guangdong, China. The goal was to provide valuable population genetic data and resources for forensic applications, supporting the “strengthen police by science and technology” initiative in China. Additionally, we investigated sequence variations in repeat regions to demonstrate the advantages of MPS technology in the application and investigation of STR genetic markers. By comparing our results with published reference populations, we systematically explored the genetic background of the Hakka people from Meizhou, Guangdong, from both biparental and paternal perspectives, offering crucial genetic evidence for analyzing the origins and migration patterns of the Hakka.

## 2 Materials and methods

### 2.1 Sample collection and DNA extraction

**FIGURE 1:**
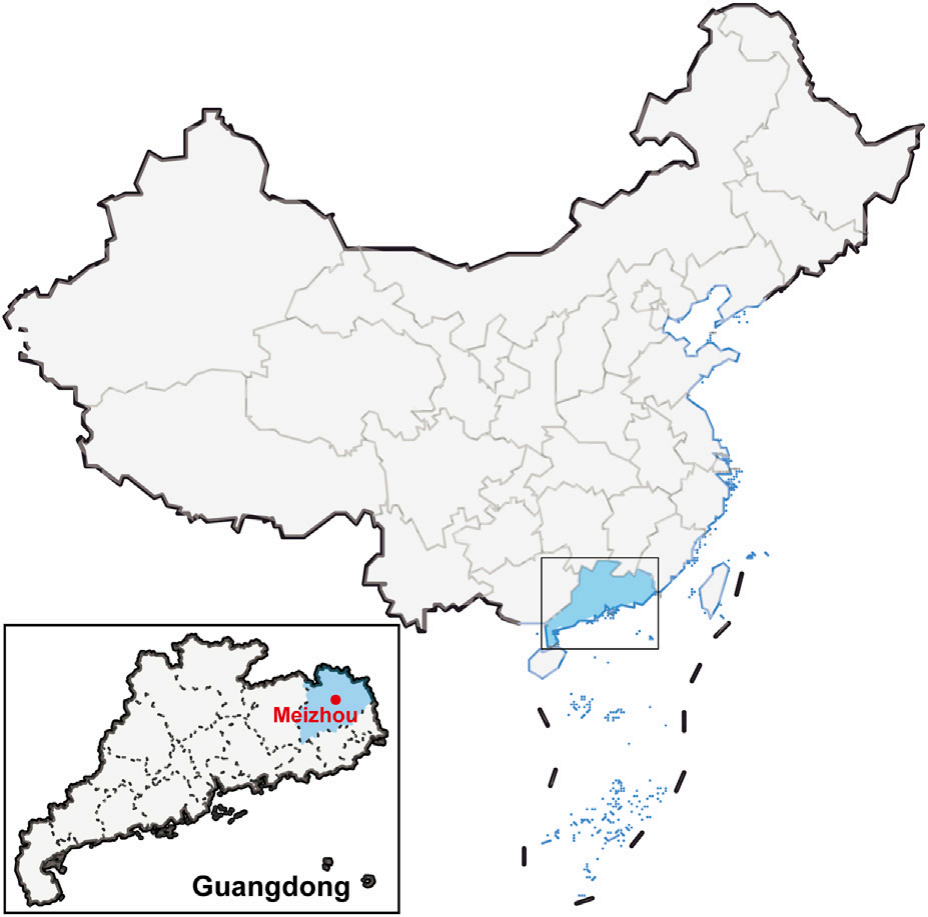
The location of sample collection of Meizhou Hakka population in Guangdong, China.

Blood samples on FTA cards were collected from 628 unrelated Guangdong Meizhou Hakka Chinese (including 547 males and 81 females) in accordance with the principle of informed consent. Ethical permission was approved by the ethics committee at the School of Life Sciences in Fudan University (Ethics Approval Document No. 14012), in accordance with the revised Helsinki Declaration of 2013. The geographical location of the research population was visualised using the “ggplot2" package in the R 4.2.3 software (https://www.r-project.org/), as can be seen in Figure 1. The sample FTA cards were pretreated with 100 μL of 0.1×TE (DeepReads Biotech, Guangzhou, Guangdong, China) and incubated at 98 °C for 30 min. The gDNA was extracted using the QIAamp^®^ DNA Blood Mini Kit (QIAGEN GmbH, Hilden, Germany), and, according to the manufacturer’s guidelines, quantification was performed using the Qubit dsDNA HS Assay Kit (Thermo Fisher Scientific, Waltham, MA, USA) applied to Qubit 4.0.

### 2.2 PCR amplification, library preparation, and sequencing

A total of 52 A- STRs (CSF1PO, D10S1248, D10S1435, D11S2368, D11S4463, D12ATA63, D12S391, D13S317, D13S325, D14S1434, D15S659, D16S539, D17S1290, D17S1301, D18S51, D18S535, D19S253, D19S433, D1GATA113, D1S1656, D1S1677, D20S470, D20S482, D21S11, D22GATA198B05, D22S1045, D2S1338, D2S441, D3S1358, D3S3045, D3S4529, D4S2366, D4S2408, D5S2500, D5S818, D6S1017, D6S1043, D6S474, D6S477, D7S1517, D7S3048, D7S820, D8S1132, D8S1179, D9S1122, D9S925, FGA, Penta-D, Penta-E, TH01, TPOX and vWA) and 81 Y STRs (DYF387S1a/b, DYF404S1a/b, DYS385a/b, DYS527a/b, DYS572, DYS19, DYS388, DYS389I, DYS389II, DYS390, DYS391, DYS392, DYS393, DYS434, DYS435, DYS437, DYS438, DYS439, DYS443, DYS444, DYS447, DYS448, DYS449, DYS450, DYS453, DYS454, DYS455, DYS456, DYS458, DYS460, DYS472, DYS476, DYS481, DYS485, DYS502, DYS505, DYS508, DYS510, DYS511, DYS512, DYS513, DYS518, DYS522, DYS530, DYS531, DYS533, DYS538, DYS541, DYS549, DYS552, DYS556, DYS557, DYS565, DYS568, DYS570, DYS571, DYS573, DYS576, DYS578, DYS585, DYS587, DYS590, DYS593, DYS596, DYS612, DYS613, DYS616, DYS617, DYS622, DYS626, DYS627, DYS630, DYS635, DYS638, DYS640, DYS641, DYS643, DYS645, DYS722, GATA A10 and GATA H4) were included in the analysis panel (Detailed information on the 133 STRs is listed in Supplementary Table S1A).

A template of 1 ng of genomic DNA (gDNA) was used, along with the Forensic Analysis System Multiplecues SetB Kit (DeepReads, Guangzhou, China), for the purpose of library construction. The PCR amplification cycle conditions were as follows: initial incubation at 98°C for 3 min; followed by 20 cycles, each consisting of 20 s at 98°C and 6 min at 60°C; concluding with extension at 72°C for 2 min; and then holding it at 4°C. The PCR products were subjected to purification using DNA Clean Beads (Enlighten, Shanghai, China). The target amplicons were encoded and amplified using the PCR cycle conditions as follows: initial incubation at 98°C for 1 min, followed by 6 cycles consisting of 20 s at 98°C, 20 s at 60°C, and 30 s at 72°C. This was followed by extension at 72°C for 2 min, and finally holding at 4°C. The library underwent purification using DNA Clean Beads and was subsequently quantified using the Qubit^®^ Fluorometer.

The barcoded DNA library is mixed in equimolar quantities and subsequently converted into a single-stranded circular DNA library through DNA denaturation and cyclization. Afterwards, using the single-stranded circular DNA as a template, DNA nanoballs (DNBs) are prepared using the rolling circle amplification (RCA) method. Subsequently, the DNBs are loaded into the flow cell using the combinatorial probe-anchor synthesis (cPAS) technique. Finally, single-end 400 nt sequencing is performed on the MGISEQ-2000 platform (MGITech, Shenzhen, China) following the manufacturer’s recommended method. The STR calling process is accomplished by adjusting the parameters of STRait Razor 3.0 (Woerner et al., 2017).

### 2.3 Statistical analysis

In this study, we initially utilised Genepop 4.7 software (Rousset, 2008) to perform the Hardy-Weinberg equilibrium (HWE) test on the population genotype data of A-STRs’ length and sequence polymorphism levels. To ensure the accuracy of the findings, a Bonferroni correction was implemented on the *p*-values. Subsequently, using the STRsAF 2.1.5 software (Gouy and Zieger, 2017), we calculated and statistically analysed forensic-related parameters for 133 STRs based on length and sequence polymorphism. Some of these parameters were expected heterozygosity (H_exp_), observed heterozygosity (H_obs_), power of discrimination (PD), exclusion probability (PE), match probability (PM), typical paternity index (TPI), and polymorphism information content (PIC), among others. In addition, the direct counting method was employed to quantify the haplotypes and their frequencies of 81 Y-STRs in the Meizhou Hakka population. Using the appropriate formulae, characteristics were calculated, such as haplotype diversity (HD), haplotype match probability (HMP), and haplotype discrimination capacity (DC) for the Y-STRs (refer to Supplementary Table S4B for the calculation formulae).

To learn more about the genetic structure of the Meizhou Hakka population, STR genotype data from 38 reference populations around the world were merged. Each of these populations had a sample size of more than 25 people. Detailed information about the populations involved in this study can be found in Supplementary Table S1B The Arlequin v3.5.2.2 software (Excoffier and Lischer, 2010) was used to calculate the *Fst* genetic distance based on A-STRs and the *Rst* based on Y-STRs among the Meizhou Hakka population and globally published reference populations. The R software (version 4.2.3) (https://www.r-project.org/) was used to visualise the two types of genetic distances generated by the two genetic markers, and heatmaps were created. Multidimensional Scaling Analysis (MDS) was performed using *Rst* as the basis, utilising IBM SPSS Statistics Version 27.0 software (Armonk, NY: IBM Corp.). The resulting data was visualised through the ggplot2 package. Phylogenetic trees by the neighbour-joining (N-J) method were constructed using MEGA 11 software (Tamura et al., 2021), using the *Fst* and *Rst* genetic matrices as the basis.

## 3 Results

### 3.1 Quality control

A total of 628 Meizhou Hakka individuals were genotyped using 133 STRs from the Forensic Analysis System Multiplecues SetB Kit. The raw data based on length polymorphism is presented in Supplementary Table S2. As shown in Supplementary Figure S1, the read depth of A-STRs fluctuates from a minimum depth of 1163.06 ± 870.908 (mean ± SD) at the D3S1358 locus to a maximum depth of 8729.4 ± 5502.351 (mean ± SD) at the D8S1132 locus. Out of the Y-STRs, the DYS572 locus had the highest read depth of 10503.84 ± 6396.134 (mean ± SD), while the DYS455 locus had the lowest read depth of 628.71 ± 512.777 (mean ± SD).

### 3.2 Allelic gene polymorphism and forensic parameters

#### 3.2.1 Allelic gene polymorphism and forensic parameters of 52 A-STRs

Supplementary Table S3A displays the allelic polymorphism and forensic-related parameters of 52 A-STRs in the Hakka population of Meizhou, Guangdong. The HWE tests were performed separately for A-STRs based on the level of sequence and length polymorphism. Prior to Bonferroni correction, we observed that the D18S535 locus deviated from HWE at both sequence and length polymorphism levels. Moreover, the D20S470 locus deviated from HWE exclusively at the length polymorphism level. Meanwhile, 11 loci (D10S1435, D22GATA198B05, D7S3048, D4S2408, D15S659, TH01, D10S1248, D22S1045, Penta-E, D9S1122, and D19S253) exhibited deviations from HWE solely at the level of sequence polymorphism. Apart from the aforementioned loci, the remaining 39 A-STRs conformed to the HWE test in terms of both sequence and length polymorphisms. After Bonferroni correction (*p* > 0.05/52), all A-STRs conformed to the HWE test.

**FIGURE 2:**
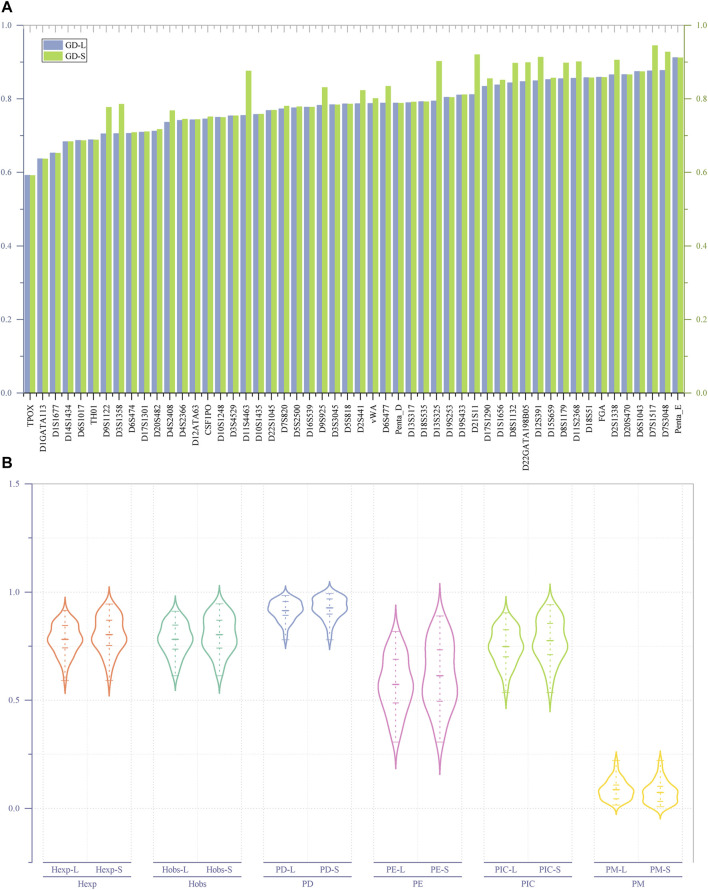
The GD values and forensic-related parameters of 52 A-STRs. **(A)** Comparison of GD values for 52 A-STRs based on repeat sequence and length polymorphisms; **(B)** Forensic parameters of 52 A-STRs. “-L” denoting length polymorphism and "-S″ indicating sequence polymorphism.

As shown in Figure 2A, irrespective of whether it is based on length or sequence polymorphism, the TPOX locus has the lowest GD value (GD _min_ = 0.59156) among the 52 A-STRs. The Penta-E locus has the highest GD value (GD-L _max_ = 0.91168) when considering length polymorphism, whereas the D7S1517 locus has the highest GD value (GD-S _max_ = 0.94487) when considering sequence polymorphism. Among the 52 A-STRs, only 15 loci exhibit no difference in GD values at the level of length and sequence polymorphism. These loci include D10S1248, D18S51, D18S535, D19S253, D1GATA113, D1S1677, D20S470, D3S3045, D5S818, D6S1017, FGA, Penta-D, Penta-E, TH01, and TPOX. The remaining loci all demonstrate differences in GD.

As illustrated in Figure 2B, for the 52 A-STRs, the H_obs_ values based on length polymorphism fluctuate between 0.61306 and 0.91083, while those based on sequence polymorphisms range from 0.61306 to 0.94586. Specifically, the TPOX locus has the lowest H_obs_ values on both length and sequence polymorphism levels, while the D7S1517 locus has the highest on both levels. The average H_obs_ values based on length and sequence polymorphisms are 0.781633 (±0.010121) and 0.802793 (±0.011699), respectively. The lowest H_exp_ values for all A-STRs based on both length and sequence polymorphisms are 0.59156, found at the TPOX locus. The highest H_exp_ value based on length polymorphism is 0.91497, found at the Penta-E locus, while the highest H_exp_ value based on sequence polymorphism is 0.94487, found at the D7S1517 locus. Statistical results showed that the average H_exp_ value on the length polymorphism level is 0.781057 (±0.009632), while on the sequence polymorphism level, it is 0.803425 (±0.011399).

The lowest PIC values based on length and sequence polymorphism were both observed at the TPOX locus; it is 0.535189. The highest PIC value based on length polymorphism was found at the Penta-E locus (0.904516), while the highest PIC value based on sequence polymorphism was observed at the D7S1517 locus (0.941391). Similarly, the lowest PE values based on length and sequence polymorphism were both found at the TPOX locus, at 0.306843. The highest PE values were all located at the D7S1517 locus, at 0.817591 and 0.889690, respectively. The combined PE (CPE) values calculated based on the PE values at each locus indicated that the CPE values at the levels of length and sequence polymorphism were 1-5.0779620E-21 and 1-3.257436E-24, respectively. All PD values of A-STRs at the levels of length and sequence polymorphism were greater than 0.778916 at the TPOX locus. The PD values based on length polymorphism were all less than 0.984178 at the Penta-E locus, while those based on sequence polymorphism were all less than 0.992419 at the D7S1517 locus. The combined PD (CPD) values observed based on length and sequence polymorphism were 1-1.727007E-59 and 1-5.517015E-66, respectively. The range of length-type PM values was between 0.015822 (Penta-E) and 0.221084 (TPOX), while sequence-type PM values fluctuated between 0.007581 (D7S1517) and 0.221084 (TPOX). The maximum TPI values for both length- and sequence-types were found at the D7S1517 locus, at 5.607143 and 9.235294, respectively. The minimum TPI values at both levels of polymorphism were found at the TPOX locus, at 1.292181.

#### 3.2.2 Genetic diversities and forensic parameters of 81 Y-STRs

**FIGURE 3:**
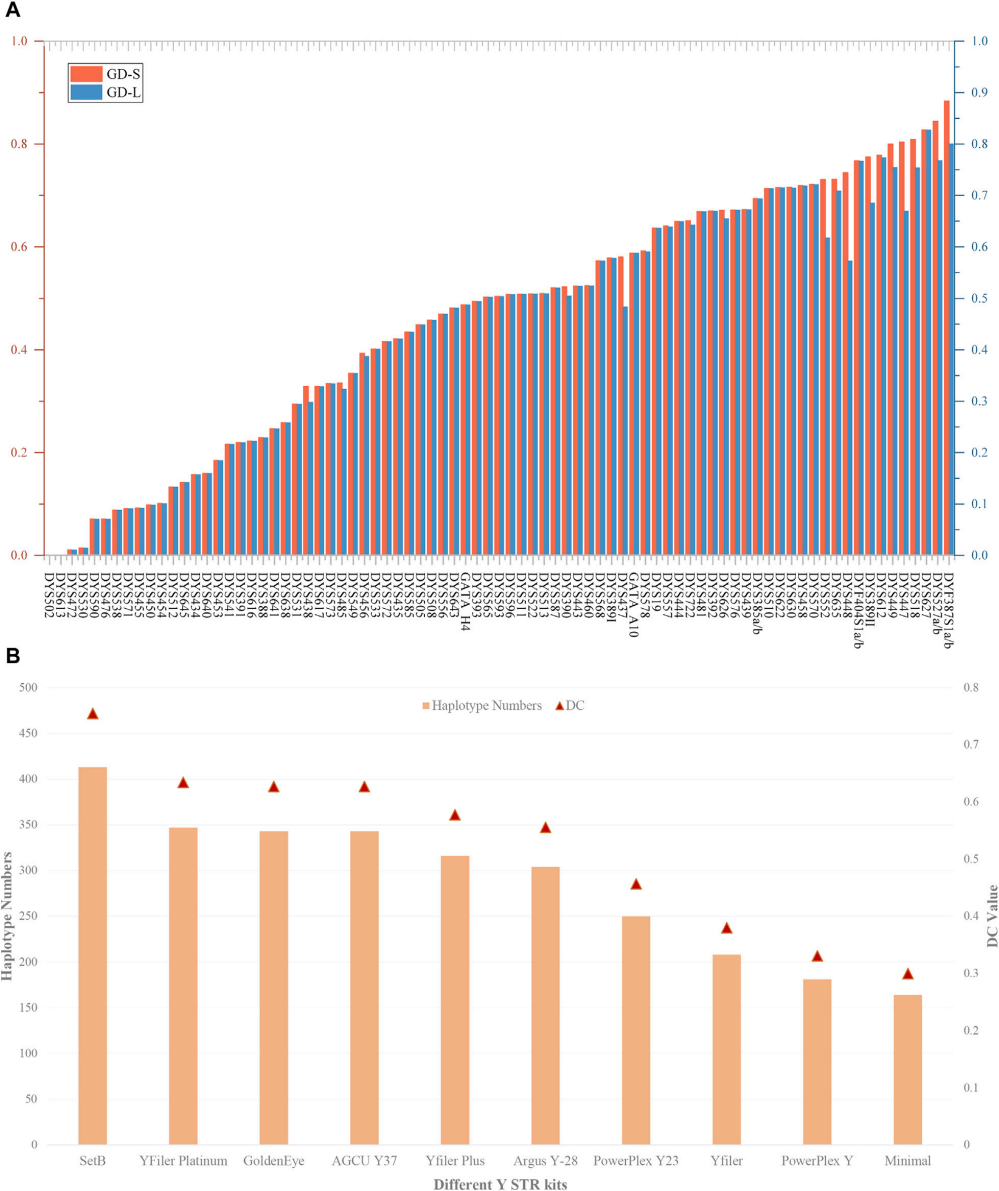
The GD values of 81 Y-STRs and different Y-STR kits in the Meizhou Hakka population. **(A)** Comparison of GD values for 81 Y-STRs based on repeat sequence and length polymorphisms; **(B)** Haplotype numbers and DC values of different Y-STR kits in the Meizhou Hakka population. The *Y*-axis on the left indicates haplotype numbers, and the *Y*-axis on the right indicates discriminatory capacity.

Supplementary Table S3B presents the haplotype results and forensic-related parameters of 81 Y-STRs at the level of length and sequence polymorphism. As illustrated in Figure 3A, apart from the GD values of loci DYS502 and DYS613 being zero, the GD values of all Y-STRs fluctuate between 0.010929 (DYS472) and 0.827879 (DYS627) based on the level of length polymorphism. On the sequence polymorphism level, the minimum and maximum GD values occur at the DYS472 and DYF387S1a/b loci, respectively, being 0.010929 and 0.884050. We discovered a discrepancy in the GD values of 22 loci at the level of length and sequence polymorphism, including DYS438, DYS485, DYS456, DYS390, DYS437, DYS578, DYS557, DYS722, DYS626, DYS630, DYS458, DYS552, DYS635, DYS448, DYF404S1a/b, DYS389II, DYS612, DYS449, DYS447, DYS518, DYS527a/b, and DYF387S1a/b.

Based on the length polymorphism of Y-STRs, 413 different haplotypes were identified in 547 Meizhou Hakka males. Of these, 79.90% were unique haplotypes. The HD, HMP, and DC values are respectively 0.99764282, 0.00418102, and 0.75502742. In contrast, based on the Y-STR sequences, 456 different haplotypes were identified, where the unique haplotypes account for 85.53%, and the HD (0.99894195) has increased, HMP (0.00288427) has decreased, and DC (0.83363803) has improved to a certain extent. To compare the forensic application efficiency of the Forensic Analysis System Multiplecues SetB Kit used in our study with other Y-STR kits, we selected nine mainstream commercial reagent kits such as GoldenEye, YFiler Platinum, AGCU Y37, Yfiler Plus, *etc.*, as references (see Supplementary Table S4A for the loci information of each reagent kit) and calculated the HD, HMP, and DC of different kits in the Meizhou Hakka population (see Supplementary Table S4B for the calculation results). As can be seen from Figure 3B, in the 547 unrelated Meizhou Hakka males, the DC values and the number of identified haplotypes of different Y-STR kits increase with the increase in the number of chosen gene loci. Meanwhile, the results showed that the 81 Y-STRs of the Forensic Analysis System Multiplecues SetB Kit have significantly higher DC and HD values (length-type alleles).

### 3.3 Sequence polymorphism analysis of STRs

Based on the MPS platform, the repeat region sequences and length polymorphisms of 133 STRs (52 A-STRs, 81 Y-STRs) were analyzed in the Meizhou Hakka population. The alleles and their corresponding frequencies are presented in Supplementary Table S5. We have observed sequence variant alleles that traditional PCR-CE could not effectively distinguish. Compared to the data generated from length polymorphism, there is a significant increase in the number of alleles detected at the level of sequence polymorphism, as detailed in Supplementary Table S6. Based solely on STR marker length polymorphisms, 1018 types of alleles were identified from 133 STRs in 628 unrelated individuals from the Meizhou Hakka population. Considering sequence variations, as illustrated in Supplementary Figure S2, the allelic diversity of 59 STRs significantly increased, resulting in a total of 1641 types of alleles identified based on sequence polymorphisms.

Specifically, based on length polymorphism, the number of alleles for 52 A-STRs is 554, with the number of alleles per locus fluctuating between 6 (D1GATA113, TPOX, and D4S2408) and 25 (Penta-E). When sequence variation was taken into account, the total number of alleles was 989, with the number of alleles fluctuating between 6 (D1GATA113 and TPOX) and 64 (D7S1517). There were 37 A-STRs that exhibit a difference in the number of alleles produced by length and sequence polymorphism; in comparison, the sequence polymorphism of the A-STRs has added a total of 435 extra alleles. The growth rate of these alleles ranges from 5.56% to 333.33%, with the number of alleles at 13 A-STR loci increasing by more than double, including D9S1122 (100.00%), D11S4463 (111.11%), vWA (125.00%), D3S1358 (157.14%), D2S1338 (207.69%), D11S2368 (209.09%), D21S11 (229.41%), D22GATA198B05 (245.45%), D13S325 (269.23%), D12S391 (315.38%), D7S3048 (316.67%), D7S1517 (326.67%), and D8S1132 (333.33%).

For 81 Y-STRs, 464 and 652 distinct alleles were identified based on length and sequence polymorphisms, respectively. There were discrepancies in the number of alleles generated by length and sequence polymorphisms at 22 Y-STR loci. Compared to length polymorphism, sequence polymorphism added a total of 188 alleles, with the growth rate fluctuating between 7.69% and 300.00%. Among them, the allele quantity growth rates for DYS527a/b (133.33%), DYS552 (150.00%), DYS449 (221.43%), DYS389II (228.575), DYS518 (233.33%), DYS447 (250.00%), and DYF387S1a/b (300.00%) all exceeded 100.00%.

### 3.4 Genetic relationship between the meizhou hakka and worldwide reference populations

#### 3.4.1 Genetic relationship among meizhou hakka and reference populations based on A-STRs

**FIGURE 4:**
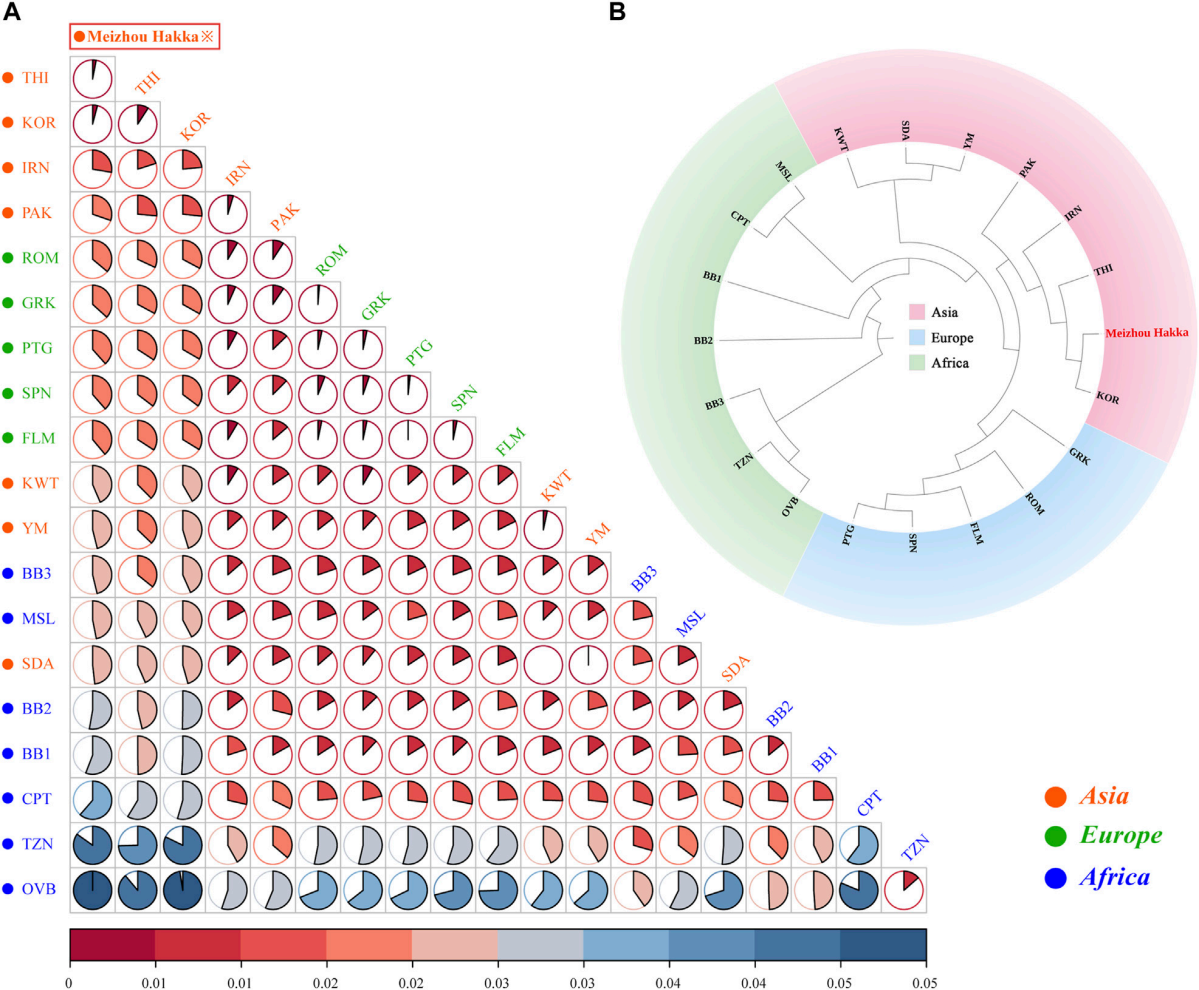
Population genetics analysis results plot based on A-STRs. **(A)** The heatmap based on the paired-*Fst* distance for Meizhou Hakka and other reference populations; **(B)** The phylogenetic tree of Meizhou Hakka and other populations based on the *Fst* genetic distances.

The length polymorphism data of 15 shared A-STRs from the Meizhou Hakka population and 19 global reference populations were merged, and the paired *Fst* values between the research population and the reference populations were calculated (see Supplementary Table S7A) and visualised through R software (Figure 4A). In the plot, the deep red represents low *Fst* values, while the deep blue indicates high *Fst* values. This is further clarified using pie charts to make the results clearer. Asian populations are denoted in red text, European populations in green, and African populations in blue. The research population is emphasised with a red border in the plot. Overall, the Meizhou Hakka population is genetically closest to the Asian populations and furthest from the African populations. Specifically, the Meizhou Hakka population is genetically closest to THI (Thai population, *Fst* = 0.001922) and KOR (Korean population, *Fst* = 0.002505), and furthest from OVB (African population, *Fst* = 0.05403).

In order to further clarify the phylogenetic relationship between the Meizhou Hakka and the global reference populations from the perspective of A-STR, we generated an N-J phylogenetic tree based on *Fst* genetic distance (Figure 4B). The research population was emphasised in bold red font in the plot and was grouped according to the continent they are located on, divided into three clusters: the Asian cluster, the African cluster, and the European cluster. From the plot, it is observed that the Meizhou Hakka population clusters with the Asian populations THI and KOR on the same branch.

#### 3.4.2 Genetic relationship among meizhou hakka and reference populations based on Y-STRs

**FIGURE 5:**
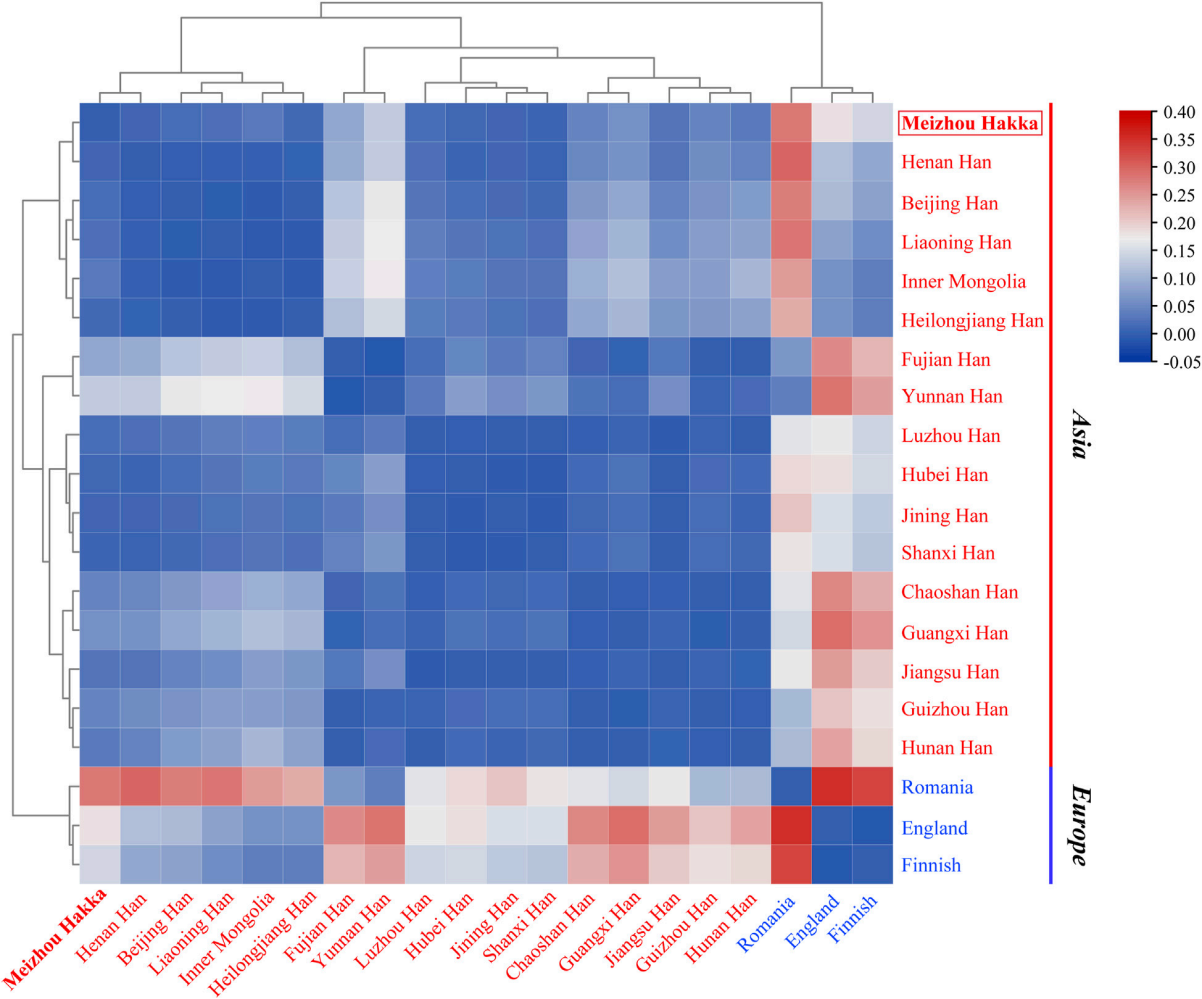
The heatmap based on the paired-*Rst* distance for Meizhou Hakka and other reference populations.

The length polymorphism data of 17 shared Y-STRs from 20 populations around the world (Meizhou Hakka and 19 reference populations) were used to caculate (see Supplementary Table S7B) the paired *Rst* values and make a heatmap (Figure 5). The deepening red colour indicates an increase in *Rst* values, while the deepening blue colour signifies a decrease. Within the heatmap, populations were marked according to their geographical locations, with red representing Asian populations and blue indicating European populations. As the plot reveals, the Meizhou Hakka maintains a relatively low genetic distance with the Han Chinese populations from Shanxi (*Rst* = 0.00832), Jining (*Rst* = 0.00895), and Henan (*Rst* = 0.00909). However, it maintains a higher genetic distance with the European populations.

**FIGURE 6:**
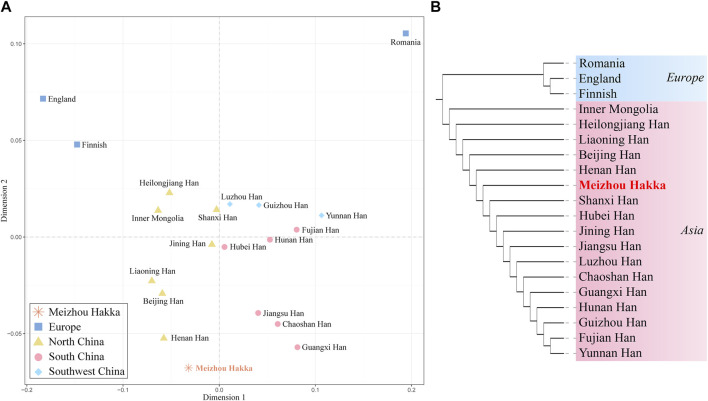
Population genetic relationship analysis results plot based on Y-STRs. **(A)** The MDS plot of Meizhou Hakka and other populations based on the paired-*Rst* distance; **(B)** The phylogenetic tree of Meizhou Hakka and other populations based on the *Rst* genetic distances.

To further verify the genetic relationship between the Meizhou Hakka and reference populations from different geographical locations, we conducted MDS analysis based on *Rst* genetic distance. As seen in Figure 6A, it roughly divides into the following four cluster groups: Two deep blue European populations are located in the top left corner of the MDS plot; the light brown Northern China populations are gathered on the left side of the plot’s centre; the centre-right is a mixed cluster of the Southwest China populations (sky blue) and some Southern China populations (peach red); part of the Southern China populations (peach red) are distributed on the lower right side of the plot. Notably, the Meizhou Hakka (represented by the asterisk and its name in bold red font) were positioned between the clustering populations of the Northern and Southern populations but are closer to the Northern populations. To analyse the genetic structure of the Meizhou Hakka population from a paternal inheritance perspective, we further constructed an NJ tree (Figure 6B) based on *Rst* genetic distance. As shown in the plot, the clustering results of different intercontinental populations are consistent with their biogeographical distribution, mainly divided into Europe (blue) and Asia (peach red). The Meizhou Hakka primarily cluster on the same branch as the Henan Han, and their upstream branches are all Northern populations, which is consistent with the conclusion obtained from the MDS plot.

## 4 Discussion

As illustrated in Supplementary Figure S1, the sequencing depth of all 133 STRs in the Meizhou Hakka individuals in our study varied significantly, ranging from 628.71 (±512.777) at the DYS455 locus to 10,503.84 (±6,396.134) at the DYS572 locus. This variation in sequencing depth across different loci demonstrates a pronounced imbalance, which may be associated with the characteristics of the specific loci (Almalki et al., 2017; Guo et al., 2017; Jager et al., 2017; Silvia et al., 2017; Xavier and Parson, 2017; Hollard et al., 2019; Fan et al., 2021). Overall, the sequencing performance of the 133 STRs included in the Forensic Analysis System Multiplecues SetB Kit met the requirements of this study.

This study conducted an in-depth analysis of the genetic polymorphism of 52 A-STRs within the Hakka population in Meizhou, Guangdong, and calculated forensic-related parameters based on both length and sequence polymorphism levels. First, after Bonferroni correction, no significant deviation from the expected HWE was observed across the 52 A-STRs. Then, as illustrated in Figure 2A, regardless of whether based on length polymorphism or sequence polymorphism, the minimum genetic diversity (GD) values were consistently found at the TPOX locus, indicating relatively low genetic diversity at this locus, which aligns with findings from previous studies (Sarkar and Kashyap, 2002; Mohapatra et al., 2004; Silva and Moura-Neto, 2004; He et al., 2017; Kim et al., 2017; Han et al., 2021; Nath et al., 2021). Conversely, the GD _max_ values based on length polymorphism were at the Penta-E, while those based on sequence information were at the D7S1517 locus. Concurrently, only 15 STRs exhibit no disparity in GD values in terms of length and sequence polymorphism, indicating that, compared to length polymorphism, sequence information may offer a more profound level of polymorphic insight.

Furthermore, as illustrated in Figure 2B, within the forensic parameters of 52 A-STRs, the average values of forensic-related parameters based on repeat region sequence exhibit higher magnitudes compared to those derived from length polymorphism, except for the PM. This indicates that considering the sequence variation in the repeat region can enhance the richness of genetic information obtained from the A-STRs. According to the “*Specification of the calculation methodology of forensic parameters for autosomal STR (SF/Z JD0105010-2018)*", the H_obs_ measure the proportion of heterozygous genotypes observed in a population for a specific genetic marker, and, in the context of HWE, the H_exp_ represents the expected proportion of heterozygous genotypes within a population. Research indicates that genetic markers with heterozygosity exceeding 0.5 are suitable for studies on genetic diversity (Davila et al., 2009). The 52 A-STRs analysed in this study yielded high H_obs_ and H_exp_ values, both at the level of length and sequence polymorphism. This suggests that these A-STRs possess significant heterozygosity, making them invaluable for forensic individual identification. And simultaneously, they provide a wealth of genetic polymorphism information for population diversity research in terms of both levels. On the other hand, studies have indicated that genetic markers with PIC values greater than 0.5 possess a high degree of informativeness, making them suitable for research into genetic features and genetic diversity (Marshall et al., 1998). We found that the PIC values of the 52 A-STRs used in our study exceed 0.5 at both levels in the Meizhou Hakka population, suggesting their applicability in forensic population genetic studies of this population. We also discovered that the CPD values observed based on sequence polymorphism are higher than those based on length polymorphism, demonstrating that A-STRs based on sequence polymorphism have greater discriminatory power in forensic individual identification. Meanwhile, incorporating repeat region sequence can enhance the efficacy of paternity testing, which is confirmed when comparing the CPE values for length and sequence polymorphism.

In our study, among the 81 Y-STRs, the use of sequence information revealed a greater diversity of haplotypes, indicating that sequence variation offers a higher DC value than length polymorphism, thus enhancing the efficacy of Y-STRs in forensic applications. At the same time, compared with other commercial mainstream Y-STR kits, the Forensic Analysis System Multiplecues SetB Kit exhibited significantly higher DC and HD values, demonstrating its superiority for forensic practice. Broadly speaking, the Forensic Analysis System Multiplecues SetB Kit showed high polymorphism and excellent discriminatory power in the Meizhou Hakka, making it suitable for forensic individual identification and paternity testing in this region. Furthermore, the forensic application of MPS technology to increase the number of genetic markers analyzed in parallel and to obtain STR genotype data based on sequence variation in repeat regions holds immense value for forensic science.

Compared to alleles characterized solely by the length of STR core repeat regions, the detection of sequence variations within these repeat regions can substantially enhance the polymorphism of these alleles. In this study, the Meizhou Hakka population data obtained from the MPS platform presented a wealth of sequence variation. Among the 133 STRs, the number of sequence-type alleles at 20 STRs has doubled or more compared to the length-type alleles; therein, the loci D3S1358, D2S1338, D21S11, D12S391, DYS389II, and DYF387S1a/b correspond to those described in the literature. Among them, the frequency of allele [AGAT]_9_, at the D17S1301 locus was 0.0263, similar to that in the Chinese population (0.0295). It may reflect the regional specificity of the allele frequencies in the STR loci (Gettings et al., 2015; Gettings et al., 2016; Novroski et al., 2016; Delest et al., 2020). Beyond these mentioned STRs, the allele count for the D8S1132 locus increased nearly 3.3 times when sequence variation was considered, illustrating that the accuracy of this locus in forensic applications may be compromised by traditional length-based analysis methods. All in all, our findings indicated that sequence variations can improve the efficiency of these STRs in forensic applications, such as individual identification and paternity testing, corroborating the conclusions of previously published research (Hussing et al., 2019; Khubrani et al., 2019; Delest et al., 2020).

We calculated the genetic distances between the Hakka and 19 reference populations, leading to a phylogenetic analysis. The findings revealed that the Hakka population has the closest genetic distance to other Asian populations, which aligns with previous research and suggests historical migrations, interactions, and genetic continuity within Asia (Tian et al., 2008). Previous studies have also suggested that most East Asian populations originated from Southeast Asia, which could explain the genetic similarities among Asian populations (Zhang et al., 2007). Additionally, we analyzed data on 17 shared Y-STRs from the 20 populations, calculating pairwise *Rst* values and conducting MDS and phylogenetic analyses. The results showed that the Meizhou Hakka population falls between the northern and southern clusters in the cluster group, but is closer to the northern cluster. This indicates that the Hakka have been genetically influenced by both northern and southern populations in China, with a stronger influence from the north, which aligns with the migratory history of the Hakka. The Meizhou Hakka population showed the closest relationship to the Henan Han population, which may be related to the origins of the Hakka. One hypothesis suggests that the Meizhou Hakka descend from migrants from the Central Plains (the ancient name for the region now called Henan in China) (Xie, 2021), and our study supports this hypothesis.

## 5 Conclusion

In conclusion, our study conducted a comprehensive analysis of length and sequence polymorphism information for 133 STRs of the Meizhou Hakka population using the MPS platform. The results indicate that sequence polymorphisms enhance the accuracy and identification efficacy of STRs in forensic case analysis, particularly in individual identification and paternity testing. Population genetic analysis suggests that the Meizhou Hakka population has been genetically influenced by both northern and southern populations, with a stronger influence from the north. The closest genetic relationship was found between the Meizhou Hakka population and the Henan Han population, supporting the “hypothesis of Descendants from Central Plains Migrants” and providing new evidence for the origins of the Hakka people. Overall, this study highlights the potential of sequence polymorphism information for improving the forensic efficacy of genetic markers and emphasizes the value of MPS in enhancing the efficiency and quality of genetic marker analysis in forensic applications. Furthermore, it offers new insights into the genetic background and historical origins of the Meizhou Hakka population, contributing to the field of population genetics research.

## Data Availability

The original contributions presented in the study are included in the article/Supplementary Material, further inquiries can be directed to the corresponding author.
