# Supplementary figures and images for: Forensic analysis and sequence variation of 133 STRs in the Hakka population
Source: Front Genet. 2024 Jan 22;15:1347868. doi: 10.3389/fgene.2024.1347868 (PMC10839782; doi:10.3389/fgene.2024.1347868)

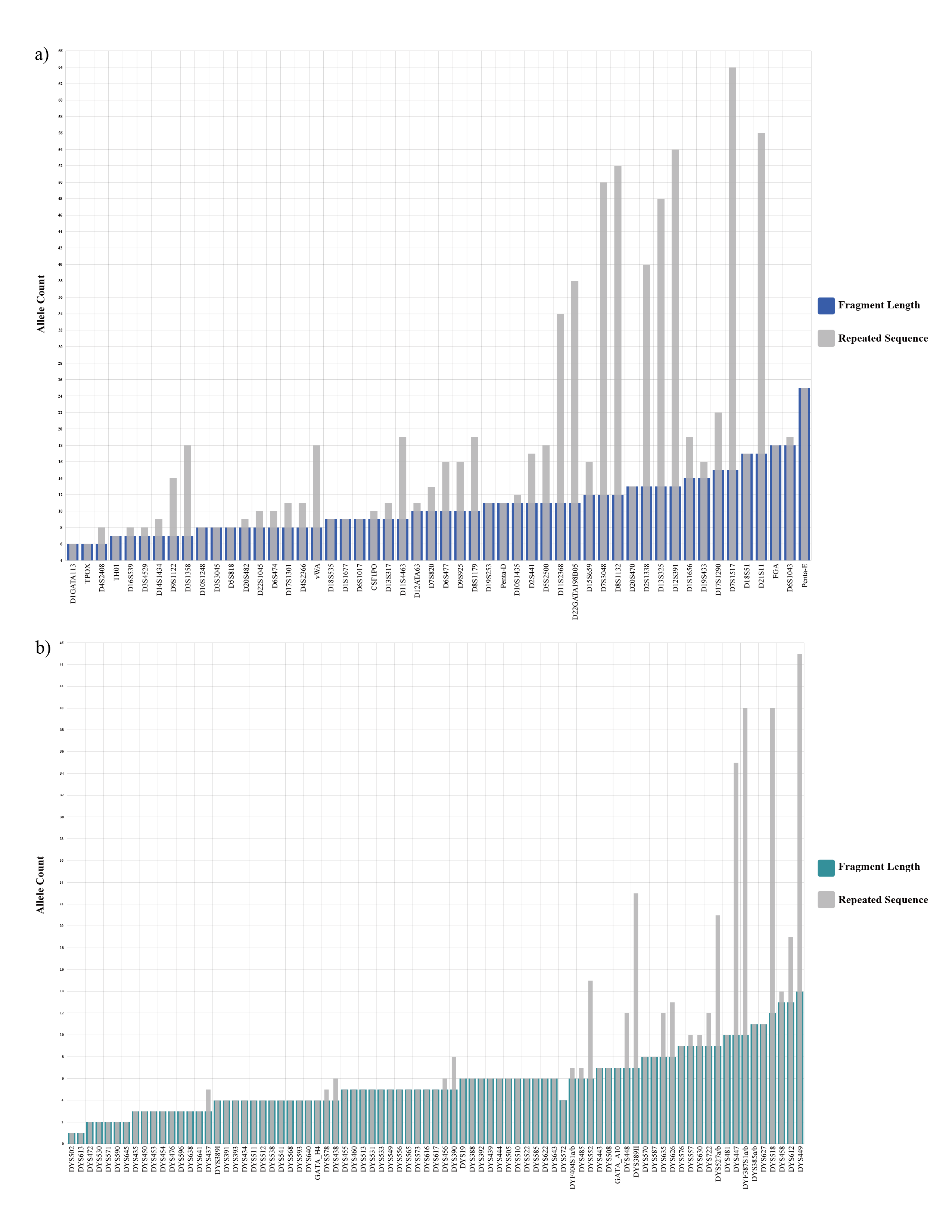

Supplement: Supplementary file 3 [file Image2.TIF]

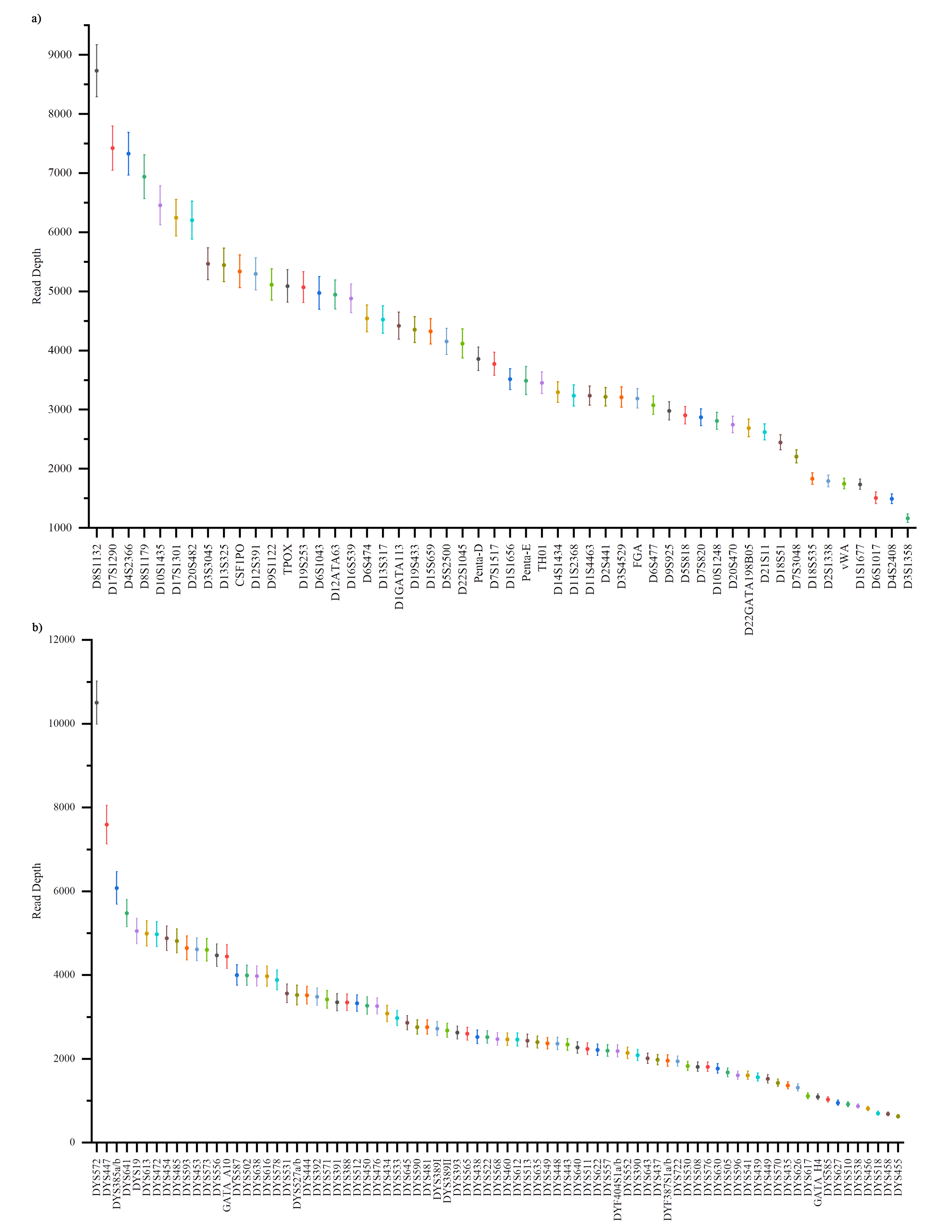

Supplement: Supplementary file 4 [file Image1.TIF]
